# Human Pirh2 is A Novel Inhibitor of Prototype Foamy Virus Replication

**DOI:** 10.3390/v7041668

**Published:** 2015-04-02

**Authors:** Lanlan Dong, Qingqing Cheng, Zhihao Wang, Peipei Yuan, Zhi Li, Yan Sun, Song Han, Jun Yin, Biwen Peng, Xiaohua He, Wanhong Liu

**Affiliations:** 1 Pathogenic Organism and Infectious Diseases Research Institute, School of Basic Medical Sciences, Wuhan University, Donghu Road No. 185, Wuchang, Wuhan 430071, China; E-Mails: donglanlan.2006@163.com (L.D.); 2012203010043@whu.edu.cn (Q.C.); wangzhihao@whu.edu.cn (Z.W.); yuanpeipei@whu.edu.cn (P.Y.); hansong@whu.edu.cn (S.H.); yinjun@whu.edu.cn (J.Y.); pengbiwen@whu.edu.cn (B.P.); 2 College of Life Sciences, Shanxi Normal University, Xi’an 710062, China; E-Mails: lizhi@snnu.edu.cn (Z.L.); sunyan@snnu.edu.cn (Y.S.); 3 Hubei Province Key Laboratory of Allergy and Immunology, School of Basic Medical Sciences, Wuhan University, Wuhan 430071, China; 4 Hubei Provincial Key Laboratory of Developmentally Originated Disease, School of Basic Medical Sciences, Wuhan University, Wuhan 430071, China

**Keywords:** PFV, Pirh2, replication, ubiquitination

## Abstract

Prototype foamy virus (PFV) is a member of the unconventional and nonpathogenic retroviruses. PFV causes lifelong chronic infections, which are partially attributable to a number of host cell factors that restrict viral replication. Herein, we identified human p53-induced RING-H2 protein (Pirh2) as a novel inhibitor of prototype foamy virus. Overexpression of Pirh2 decreased the replication of PFV, whereas knockdown of Pirh2 with specific siRNA increased PFV replication. Dual-luciferase assays and coimmunoprecipitation demonstrated that Pirh2 negatively influences the Tas-dependent transcriptional activation of the PFV long terminal repeat (LTR) and internal promoter (IP) by interacting with the transactivator Tas and down-regulating its expression. In addition, the viral inhibitory function of Pirh2 is N-terminal and RING domain dependent. Together, these results indicated that Pirh2 suppresses PFV replication by negatively impacting its transactivator Tas and the transcription of two viral promoters, which may contribute to the latency of PFV infection.

## 1. Introduction

Prototype foamy virus (PFV), first known as human foamy virus (HFV), is a member of foamy viruses (FVs), which was originally isolated from a nasopharyngeal carcinoma patient [1]. Several decades of researches revealed no association of FV infection with any clinical symptom in accidentally infected humans, and FVs can maintain a lifelong infection in the host [2,3,4]. Several cellular genes have been identified that actively restrict PFV replication by targeting distinct steps in the viral life cycle. For example, APOBEC3C acts predominantly on viral nucleic acids [5,6], TRIM5α blocks viral DNA synthesis and intracellular trafficking [7,8], and tetherin inhibits prototypic foamy virus release [9,10]. All of those cellular factors may contribute to lifelong persistent infections of PFV.

As we know, foamy viruses are unique retroviruses that are significantly different from conventional retroviruses in genomic structure and replication strategy [11,12]. The provirus not only contains the classical retroviral long terminal repeat (LTR) promoter for the three retroviral structural genes group specific antigen (*gag*), polymerase (*pol*), and envelope (*env*), but it also has a second functional internal promoter (IP) within the *env* gene directing the synthesis of the transactivator *tas* and the accessory gene *bet* [13]*.* The regulation of these two promoters’ transcription activities was shown to be the key to determining whether the FV infection is lytic or persistent [14]. Because both the LTR promoter and the IP are efficiently transactivated by Tas and transcription activity of LTR promoter is strictly Tas-dependent [15], Tas acts as a key factor to determine the latent or lytic replication of FVs [14,16]. It has been reported that proteins, such as PML, N-Myc interactor (Nmi), and PCAF, can interact with Tas and further influence the viral transcription through different mechanisms [17,18,19]. Thus, to find a protein that affects PFV replication, we attempted to investigate the Tas-interacting proteins.

Herein, we identified a novel cellular factor by screening a human 293T cDNA library using the yeast two-hybrid assay with Tas as the bait. Human p53-induced RING-H2 protein (Pirh2), which is an E3 ubiquitin ligase and mediates ubiquitin-proteasome degradation of proteins [20], was determined to be a Tas-interacting protein. Moreover, we demonstrated that overexpression of Pirh2 led to the inhibition of PFV replication, and knocking down Pirh2 increased viral gene expression. In addition, Pirh2 acted to repress the Tas-dependent transcriptional activation of the viral LTR and IP promoters and down-regulate Tas expression through the ubiquitin-degradation pathway.

## 2. Materials and Methods

### 2.1. Cell Culture, Reagents, Antibodies

The 293T cells and PFV indicator cell line (BHK21-derived indicator cells encoding a luciferase gene driven by the PFV LTR promoter) were grown in Dulbecco’s modified Eagle medium (DMEM) supplemented with streptomycin (100 μg/mL), penicillin (100 U/mL), and 10% (vol/vol) fetal bovine serum (FBS) in petri dishes at 37 °C in a humidified atmosphere containing 5% CO_2_. All cell culture reagents and media were purchased from Hyclone (Hyclone Laboratories, Logan, UT, USA). MG132 (S2619) was purchased from Selleck (Selleck Chemicals, Houston, TX, USA), Anti-myc (2276S), anti-Flag (8146S), anti-HA (3724S), and anti-His (2366S) were from Cell Signaling Technology (CST). Antibody against β-actin (ab3280) was obtained from Abcam (Cambridge, UK), and anti-Pirh2 (BL588) was from Bethyl. Antibody against PFV Gag was kindly provided by Professor Li Zhi, and anti-Tas was produced by immunizing rabbits with prokaryotic expressed Tas and purified according to standard procedures [21]. HRP-conjugated goat anti-rabbit or goat anti-mouse secondary antibodies were from Proteintech.

### 2.2. Plasmids and siRNA and Transfection

Plasmids LTR-Luc, IP-Luc, myc-Tas and TK-Tas were constructed based on the infectious pHSRV13 provirus DNA, which was a gift from Professor Rolf M. Flügel (German Cancer Research Center) [22]. The fragments LTR (from −533 to +20) and IP (from 8971 to 9438) were amplified from pHSRV13 and inserted into promoterless luciferase reporter construct pGL3 (Promega, Fitchburg, WI, USA) [14,23]. pHSRV13 was also used as the template for amplifying the entire *tas* gene, and the fragment was inserted into pCMV-myc and pEGFP-C1. TK-Tas was generated by replacing the RLuc gene of pRL-TK (provided by professor Liu Xin, Wuhan University) with *tas* gene. Flag-Pirh2 and pDsRed-n1-Pirh2 were constructed by using cloned full length Pirh2 gene into a eukaryotic expression vector pCMV-3Tag or pDsRed-n1 from cDNA 293T cells. The three individual domains, N terminal domain (NTD, residues 1–137), RING domain (R, residues 138–189), and N terminal domain (CTD, residues 137–261), and three mutants, which deleted one of the three domains, were PCR amplified and cloned into pCMV-3Tag [24]. c-MYC was kindly provided by professor Lou Zhenkun and HA-Ub was gift from professor Shu Hongbing. All the primers used for plasmid construction were aligned in Table S1 in the Supplementary Material. pGEX-4T-1-Tas (GST-Tas), MBP-Pirh2 and His-Gag were kept by our laboratory. Pirh2-specific siRNA (5'-CAUGCCCAACAGACUUGUG-3') (target site from 207 to 225) and a nonsilencing siRNA (NC) (used as a negative control) were purchased from (GenePharma Shanghai, China) [25,26]. Plasmids and siRNA transfections were performed by using lipofectamine 2000 reagent (Life Technologies, Grand Island, NY, USA) according to the manufacturer’s instructions.

### 2.3. Yeast Two-Hybrid Screening

Yeast two-hybrid screening was conducted according to the manufacturer’s instructions with Matchmaker GAL4 Two-Hybrid System 3 (Clontech, Mountain View, CA, USA). In brief, the fragment encoding the full-length Tas was generated by PCR cloned into pGBKT7 (Clontech) and used as the bait. A human 293T cDNA library was used as the prey. Then, AD and BD vectors were co-transformed into the yeast strain AH109 and used to screened the positive clones on synthetic dropout medium lacking tryptophan, leucine, and histidine (SD/-Trp/-Leu/-His) or tryptophan, leucine, histidine, and adenine (SD/-Trp/-Leu/-His/-Ade). Subsequently, the co-transformants were assayed for α and β-galactosidase activity to further identify the positive clones.

### 2.4. Virus Preparation and Infection

PFV was prepared by transfecting 293T cells with infectious pHSRV13 provirus DNA by the modified calcium phosphate method [22,27]. After 48 h, the transfected cells and the culture medium were frozen and thawed for three cycles to release virus. To prepare cell-free virus stocks, the culture supernatants were centrifuged at 4000× *g* for 10 min and filtered through a 0.22 μm pore size filter membrane, and stored at −80 °C. PFV titers were determined by infecting PFV indicator cells [23]. Before infection, the 293T cells medium was removed and the cells were incubated with a dilution of virus (multiplicity of infection of 0.1) for 1.5 h in an incubator. Then, the supernatant was replaced with growth medium and maintained for 48 h.

### 2.5. Coimmunoprecipitation and Western Blot

For analysis of protein-protein interactions, 1 × 10^7^ 293T cells were seeded in 10 cm dish and then were transfected with myc-Tas (12 μg) and Flag-Pirh2 (12 μg). After 24 h, cells were washed twice with ice-cold PBS and disrupted in IP lysis buffer (Beyotime, Shanghai, China) with 1 mM PMSF and protease inhibitor cocktail tablets (Roche, Branchburg, NJ, USA). Cell lysates were centrifuged at 12,000 rpm for 10 min at 4 °C. Then, the supernatants were incubated with the indicated antibody for 4 h at 4 °C. Next, Protein A/G plus-Agarose (Santa Cruz, Berkeley, CA, USA) was added to the mixture, which was rotated overnight at 4 °C. The agarose was washed five times with lysis buffer, resuspended in 20 μL lysis buffer and 20 μL 2× SDS loading buffer and boiled for 5 min. The protein samples were then subjected to Western blot. Equal amounts of protein extracts or immunoprecipitated materials were separated using 10% sodium dodecyl sulfate-polyacrylamide gel electrophoresis (SDS-PAGE) and transferred to polyvinylidenedifluoride (PVDF) membrane (Roche). The membranes were blocked in 5% nonfat milk-TBST for 2 h at room temperature and probed with the indicated primary antibodies overnight at 4 °C followed by washing with 1× TBST for 10 min × 3 times. Then, the membranes were hybridized with HRP-conjugated goat anti-rabbit or goat anti-mouse secondary antibodies for 1.5 h at room temperature and washed three times with 1× TBST for 15 min. The reacting bands were visualized with the enhanced chemiluminescence (ECL) system (Advansta, Menlo Park, CA, USA) and X-ray film. The quantitative analysis of the relative intensities of proteins (normalized to β-actin) was performed with Quantity One Software (Bio-Rad, Hercules, CA, USA) and GraphPad Prism 5.

### 2.6. Dual-Luciferase Reporter Assay

An amount of 2 × 10^5^ 293T cells were seeded into a 24-well culture plate and transfected with PFV LTR-Luc (40 ng) or IP-Luc (20 ng) with or without TK-Tas (50 ng), together with Flag-Pirh2 (400 ng) plasmid or pCMV-Flag (400 ng) (as a control), and RL-TK (5 ng) was used to normalize the transfection efficiency using lipofectamine 2000. For the foamy virus activated luciferase (FAL) assay, Flag-Pirh2 or different Pirh2 mutations were transfected into 293T cells for 24 h, pCMV-Flag was transfected as a control, and the cells were challenged with PFV at a multiplicity of infection (MOI) of 0.1 for another 48 h, then those infected 293T were incubated with a PFV indicator cell line the (PIC) for 48 h, and RL-TK was transfected into PIC as an internal control 12 h before incubation. Thereafter, the firefly and Renilla luciferase activities were determined using the Dual-Glo luciferase assay (Promega) according to the manufacturer’s instructions. All transfections were performed in sextuplicate.

### 2.7. Quantitative Reverse Transcriptase Polymerase Chain Reactions

First, 1 × 10^6^ 293T cells were seeded in 6-well plate, and 18 h later cells were transfected with Flag-Pirh2 (4 μg) or Pirh2-siRNA (100 pmol) for 24 h (pCMV-Flag (4 μg) or NC (100 pmol) was used as a control) and then challenged with PFV for another 48 h. Then, cells were harvested and total RNA was extracted with Trizol reagent (Invitrogen, Carlsbad, CA, USA) by referring to the RNA Isolation Procedure provided by the Life technologies and 2 μg total RNA were reverse transcribed using the Revert Aid™ First Strand cDNA Synthesis Kit (Thermo Scientific, Rockford, USA) according to the manufacturer’s protocols. Expression levels of *gag* and *tas* were analyzed by real-time quantitative PCR (RT-qPCR) using SYBR Green PCR master mix and iCycler thermal cycler PCR (Bio-Rad). Real-time qPCR was carried out with 1μL cDNA, 0.5 μL forward primers, 0.5 μL reverse primer, and 10 μL All-in-One^TM^ qPCR mix (Gene Copoeia, Rockville, MD, USA city, state if US, country) diluted to a 1× concentration in a final volume of 20 μL. PCR was performed using the following conditions: 95 °C for 5 min, followed by 40 cycles of 95 °C for 15 s, 58 °C for 20 s, and 72 °C for 15 s. The primer sequences used in the experiment are as follows: 5'-AATAGCGGGCGGGGACGACA-3' and 5'-ATTGCCACGCACCCCAGAGC-3' for *gag*, as the primers are localized downstream of the pol splice acceptor, the products also include *pol* transcript [11,13], 5'-GGAACAATCAGATACTGACCCT-3' and 5'-CCAACTTCAGGATCCCATCTT-3' for *tas* the products of *tas* primers include the genomic, *pol*, *env* and *tas* mRNAs, 5'-CACGATGGAGGGGCCGGACTCATC-3' and 5'-TAAAGACCTCTATGCCAACACAGT-3' for β-actin. The relative quantification was employed and the 2^−ΔΔCT^ method was used to calculate the relative mRNA expression.

### 2.8. Cellular Ubiquitination Assay

A total of 1 × 10^7^ 293T cells were seeded in 10 cm dish and then were transfected with HA-tagged ubiquitin (HA-Ub) (4 μg), myc-Tas (8 μg) or c-MYC (8 μg), with Flag-Pirh2 (8 μg) or pCMV-Flag (8 μg) as a negative control, and Pirh2 polyubiquitinating c-MYC used as the positive control [28]. After 24 h, the cells were washed with ice-cold phosphate-buffered saline and collected with 1 mL IP lysis buffer (Beyotime) with 1 mM PMSF and lysed at 4 °C for 30 min. Soluble protein fraction was separated by centrifugation at 12,000 rpm for 10 min, and 1 mg whole cell protein was immunoprecipitated with 2 μg anti-myc antibody for myc-Tas or 2 μg anti-His antibody for His-c-MYC, for 4 h and then incubated with 40 μL Protein A/G plus-Agarose per sample overnight. The beads were washed with IP buffer 5 times and resuspended in 20 μL lysis buffer and 20 μL 2× SDS loading buffer and boiled for 5 min. The immunoprecipitate was analyzed with anti-HA antibody by Western blot. The whole cell lysate was assessed with anti-His, anti-Flag, and anti-myc by Western blot.

### 2.9. Purification of Recombinant Proteins and GST Pull-Down Assay

MBP-Pirh2 and GST-Tas were expressed in *Escherichia coli* (BL21) cells and purified from the bacterial. The protocol of purification Tas was described previously [21]. Additionally, MBP-tagged Pirh2 was induced expression by 0.1 mM isopropyl β-D-thiogalactopyranoside (IPTG) at 30 °C for 2 h. The cells were lysed in ice-cold bacterial lysis buffer (1 mM EGTA, pH 8.0, 100 mM KCl, 50 mM Tris-HCl, pH 8.0, 1 mM EDTA, pH 8.0 and 1 M NaCl. Adding the 0.5 mM DTT, 1 mM PMSF, and 1× Protease Inhibitor Cocktail before use). Additionally, sonicating the cells until suspension is clear. The cell extracts were incubated with amylose resin (New England Biolabs, Ipswich, MA, USA) at 4 °C for 2 h. The MBP fusion protein-bound amylose resins were washed three times with wash buffer (1 mM EGTA, pH 8.0, 200 mM KCl, 50 mM Tris-HCl, pH 8.0, 1 mM EDTA, pH 8.0, 10% (v/v) Glycerol, adding the 0.5 mM DTT, 1 mM PMSF, and 2 mM Na_2_S_2_O_5_ before use). Then, MBP-Pirh2 were eluted with the wash buffer 10 mM Maltose was added. Recombinant purified 500 µg MBP-Pirh2 was incubated with 500 µg GST-Tas or GST in 2mL assay buffer (4.2 mM Na_2_HPO_4_, 2 mM KH_2_PO_4_, 140 mM NaCl, 10 mM KCl) at 4 °C overnight. Then proteins were incubated with the 100 μL of 50% slurry of glutathione-Sepharose beads (GE Healthcare, Bucks, UK) for an additional 2 h. The mixture was extensively washed with the assay buffer. Bound proteins were eluted with 50 μL 30 mM reduced glutathione and then add 50 μL 2× SDS loading buffer to boil for 10 min. 10 μL aliquots of each sample used to run the SDS-gel and detected by Western blot.

### 2.10. Statistical Analysis

All dual-luciferase reporter assay experiments were performed in sextuplicate and repeated three times. The RT-qPCR and Western blot were independently repeated three times. The results are presented as mean ± standard deviation (SD). To test for statistical significance, a Student’s *t* test was applied. A *p* value of <0.05 was considered statistically significant.

## 3. Results

### 3.1. Tas Interacts with Pirh2

**Figure 1:**
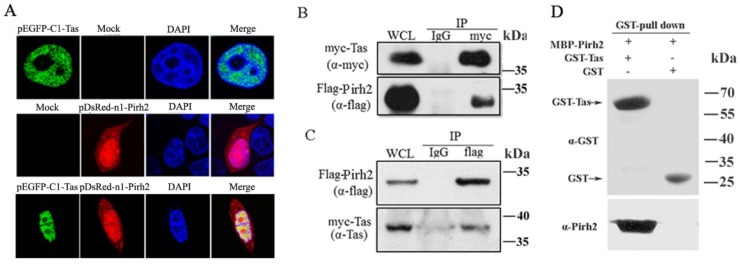
Tas interacts with Pirh2. (**A**) Tas colocalized with Pirh2 in nucleus. pEGFP-C1-Tas or/and pDsRed-N1-Pirh2 were transfected into 293T cells, 24 h after transfection, nuclei were visualized with DAPI staining; (**B**) Co-IP of Tas and Pirh2. 293T cells were cotransfected with myc-Tas and Flag-Pirh2, 24 h post transfection, the whole cell lysate was harvested and Tas was immunoprecipitated with anti-myc and normal IgG was used as negative control, then the immunoprecipitates were detected by Western blot using anti-myc and anti-Flag; (**C**) Reciprocal Co-IP of Tas and Pirh2. Immunoprecipitation (IP) was performed with anti-Flag, and analyzed by Western blot using anti- Flag and anti-Tas; (**D**) *In vitro* interaction between Tas and Pirh2. The purified Pirh2 protein was incubated with GST or GST-Tas agarose beads. GST pull down was then performed, and precipitates were detected by Western blot using an anti-Pirh2 antibody. Purified GST and GST-Tas used in the pull-down assay detected α-GST.

To screen proteins that may impact PFV replication through interaction with Tas, a human 293T cDNA library was screened in a yeast two-hybrid assay with Tas as the bait. We obtained 25 positive clones, and one of them contained a 786 bp cDNA fragment (GenBank accession No. GQ250944.1) encoding the full length protein Pirh2 and confirmed the interaction by yeast two-hybrid assay. To validate their interaction, we first detected the subcellular locations of Tas and Pirh2 by transfecting pEGFP-C1-Tas or pDsRed-N1-Pirh2 into 293T cells for 24 h, to investigate whether they would change the distribution by co-expressing with each other, 293T cells were also cotransfected with pEGFP-C1-Tas and pDsRed-N1-Pirh2 for 24 h, and examining the transfected cells with an Olympus confocal microscope. The distribution of Tas was almost completely nuclear, while, Pirh2 was distributed throughout both the nucleus and the cytoplasm, as reported previously [29], and they colocalized predominantly in the nucleus and the co-expression did not change their distribution in the cells (Figure 1A). To further confirm the interaction between Tas and Pirh2, coimmunoprecipitation was performed by co-transfecting 293T cells with myc-Tas and Flag-Pirh2. After 24 h, the cell lysate was divided into two equal portions and immunoprecipitated with anti-myc or with normal IgG as a control. Then, the precipitates were assessed by Western blot using anti-myc and anti-Flag antibodies. The data showed that Flag-Pirh2 could be coimmunoprecipitated with Tas (Figure 1B). Tas was consistently coimmunoprecipitated with Flag-Pirh2 (Figure 1C). In order to determine whether Tas binds directly to Pirh2, we expressed MBP-Pirh2 and GST-Tas in *E. coli* (BL21) cells and purified the two proteins. Then we performed Glutathione S-transferase (GST) pull-down assays using purified GST-Tas and MBP-Pirh2. The results indicated Pirh2 was eluted together with GST-Tas but not GST, suggesting a direct association of Pirh2 and PFV Tas (Figure 1D). Taken together, these results suggested that Pirh2 was able to interact with Tas.

### 3.2. Pirh2 Inhibits PFV Replication

**Figure 2:**
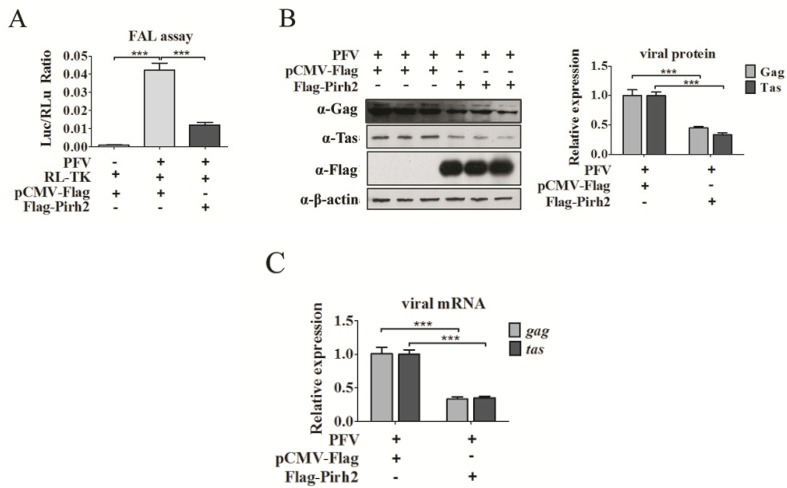

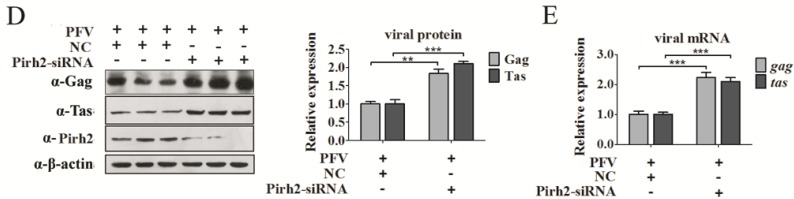
Pirh2 inhibits the replication of PFV. (**A**) The relative viral load in the presence or absence of overexpressed Pirh2 was analyzed using the FAL assay with a PFV indicator cell line. RL-TK (5 μg) was transfected as an internal control; (**B**) 293T cells that seeded in 6-well plate and challenged with PFV (MOI 0.1) were transfected with Flag-Pirh2 (4 μg) or pCMV-Flag (4 μg) (as a control), the level of the viral protein Gag and Tas were analyzed by Western blot with β-actin used as a loading control. Quantitation of Gag and Tas protein levels from the Western blot by using Quantity one software (Bio-Rad); (**C**) 293T cells were seeded in 6-well plate and transfected with Flag-Pirh2 (4 μg) for 24 h (pCMV-Flag (4 μg) was used as a control) and then challenged with PFV for another 48 h. Then, the total RNA (2 μg) was reverse transcribed to cDNA. RT-qPCR was used to examine the relative expression (normalized to β-actin) of the viral structural gene *gag* and the regulatory gene *tas* with or without Pirh2 overexpression; (**D**) Specific siRNA used to knockdown Pirh2 (100 pmol) and NC (100 pmol) used as a negative control. The viral protein Gag and Tas was detected by Western blot, quantitation analysis of Gag and Tas intensity from the Western blot by using Quantity one software (Bio-Rad); (**E**) Relative mRNA expression (normalized to β-actin) of the viral structural gene *gag* and regulatory gene *tas* in cells that were transfected with Pirh2 siRNA or NC were assessed by RT-qPCR. All the data are representative of three independent experiments with triplicate samples. (Student’s *t*-test, ** *p* < 0.01; *** *p* < 0.001).

Because Pirh2 was able to interact with Tas, we evaluated whether Pirh2 was able to affect PFV replication. First, we determined whether the cellular PFV viral load would change with Pirh2 overexpression. For relative viral load detection, the PFV-infected 293T cells (1 × 10^4^) were incubated with a PFV indicator cell line (PIC) (1 × 10^5^) for 48 h [19]. The PIC was established by stably transfecting baby hamster kidney-21 (BHK-21) cells with a luciferase gene driven by the PFV LTR and was more sensitive for quantifying PFV than TCID_50_ [23]. Twelve hours before incubating with the infected 293T cells, the PIC was transfected with RL-TK plasmid expressing Renilla (RLu) luciferase as an internal control. After 48 h, the luciferase activity was assessed. The FAL assay results (Figure 2A) showed that PFV was able to more prominently activate the PIC compared to the control group, and the Luc/RLu ratio of Flag-Pirh2-transfected cells was significantly reduced compared to what was observed in pCMV-Flag-transfected cells (Figure 2A), suggesting that overexpressed Pirh2 decreased virus production. Second, Western blot was performed to analyze the cellular viral proteins Gag and Tas in PFV-infected cells with or without Pirh2 overexpression. As shown in Figure 2B, Pirh2 overexpression led to an evident reduction in the Gag and Tas proteins of over 60% compared to the control, demonstrating that Pirh2 overexpression inhibited PFV gene expression. Third, to investigate whether Pirh2 affects the viral transcription, RT-qPCR was performed to evaluate the relative mRNA levels of the PFV structural gene *gag* and the regulatory gene *tas* in PFV-infected 293T cells with or without Pirh2 overexpression. These results showed that with overexpression of Pirh2, the viral *gag* and *tas* mRNAs were markedly decreased compared to the control (Figure 2C), suggesting that Pirh2 may have an influence on transcription during an early stage of viral replication.

To further confirm the inhibition of endogenous Pirh2 for PFV, we utilized a gene-specific siRNA to knockdown Pirh2 in 293T cells. 293T cells were transfected with either Pirh2-siRNA or NC (used as a negative control) for 24 h and challenged with PFV for another 48 h [30]. The knockdown efficacy of endogenous Pirh2 protein by Pirh2-siRNA was confirmed by Western blot. As expected, siRNA down regulated Pirh2 protein level effectively and the loss of Pirh2 resulted in enhanced PFV replication as evidenced by facilitated production of the viral structural protein Gag (Figure 2D) and the regulatory protein Tas (Figure 2D), as assessed by the Western blot method. The mRNA of the viral genes *gag* and *tas* were consistently increased by over two-fold in the RT-PCR analysis when Pirh2 was knocked down (Figure 2F). Collectively, our results suggest for the first time that host cell endogenous Pirh2 is a novel PFV replication inhibitor.

### 3.3. Pirh2 Negatively Regulates the Tas-Dependent Transcriptional Activation of PFV LTR and IP

**Figure 3:**
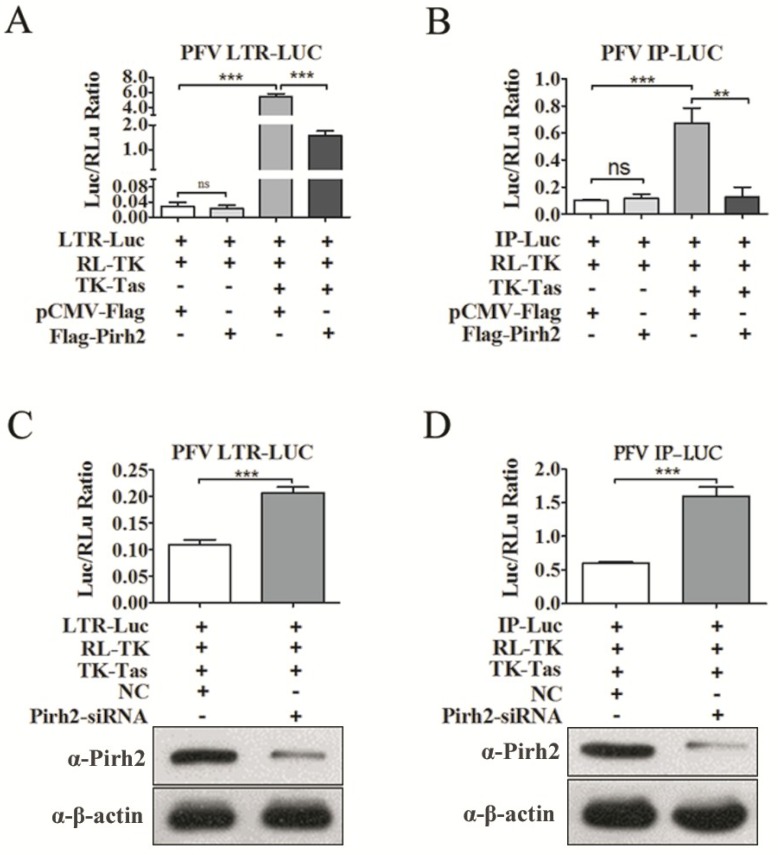
Pirh2 negatively regulates the Tas-dependent transcriptional activation of PFV LTR and IP. (**A**,**B**) Overexpressing Pirh2 down-regulates Tas-dependent transcription of the PFV LTR and IP. 293T cells were transfected with LTR-Luc (40 ng) (**A**) or IP-Luc (20 ng) (**B**) in the presence or absence TK-Tas (50 ng) combined with Flag-Pirh2 (400 ng) or pCMV-Flag (400 ng) (as a control); RL-TK was used to normalize transfection efficiency. At 48 h post transfection, luciferase activities were measured according to the manufacturer’s instructions; (**C**,**D**) Knockdown of Pirh2 with its specific siRNA up-regulates Tas-dependent transcription of the PFV LTR and IP. 293T cells were transfected with LTR-Luc (**C**) (40 ng) or IP-Luc (20 ng) (**D**) in the presence TK-Tas (50 ng) combined with Pirh2-siRNA (20 pmol) or nonsilencing siRNA NC (20 pmol) (as a negative control); RL-TK (50 ng) was used to normalize transfection efficiency. At 48 h post transfection, luciferase activities were measured according to the manufacturer’s instructions. (Student’s *t*-test, ** *p* < 0.01; *** *p* < 0.001).

Given that Pirh2 repressed PFV at both the protein and mRNA levels, this may imply that Pirh2 may function at the transcription stage or an early stage of the virus life cycle. Because many other factors that interact with Tas may influence the transactivity of Tas, we speculated that Pirh2 may suppress viral transcription. To investigate whether Pirh2 interfered with the basal or Tas-dependent transcriptional activation of both the conventional LTR promoter and IP, a luciferase reporter gene under the control of the PFV LTR (LTR-Luc) or the IP (IP-Luc) was utilized to assess the influence of Pirh2 on PFV gene transcription. We transfected 293T cells with PFV LTR-Luc or IP-Luc, with or without TK-Tas, together with Flag-Pirh2 plasmid or pCMV-Flag as a control, and RL-TK was used to normalize the transfection efficiency. Forty-eight hours post-transfection, Luc/RLu activity was measured. We observed that Tas dramatically activated theg LTR (Figure 3A) and IP (Figure 3B) as expected (Figure 3A,B). Pirh2 did not impact the basal transcriptional activation of neither promoter (Figure 3A,B). However, Pirh2 significantly impaired the Tas-dependent transcriptional activation of the LTR and IP. As shown in Figure 3A,B, with overexpressed Pirh2, Tas transactivation of the LTR and IP was inhibited over 70% compared to the control. In contrast, Pirh2-specific siRNA transfection resulted in observably elevated Tas-dependent transcriptional activation of both the LTR (Figure 3C) and the IP (Figure 3D) compared to the control, the knockdown efficacy of Pirh2 protein by Pirh2-siRNA was confirmed by Western blot. These results indicated that Pirh2 could act as a repressor of Tas-dependent transactivation of the PFV LTR and IP. However, whether Pirh2 had negative effect on the PFV uncoating, entry or integration remained uncertain. Affecting one of those stages are sufficient to impact the viral protein and mRNA levels. Thus, we could not exclude that Pirh2 may influence the cellular uncoating-triggering signals, or Pirh2 may interfere the receptors on the cell membrane for PFV entry, and even affect the integration process. Further research is required.

### 3.4. The Pirh2 N-Terminal Domain and RING Domain are Responsible for Its Inhibitory Effect on PFV Transcription and Replication

**Figure 4:**
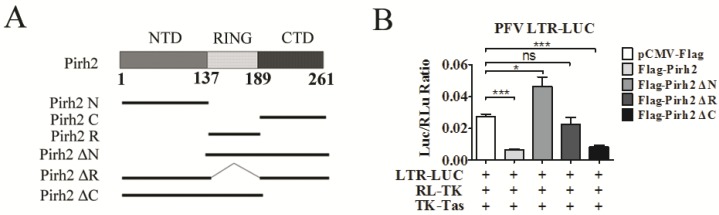

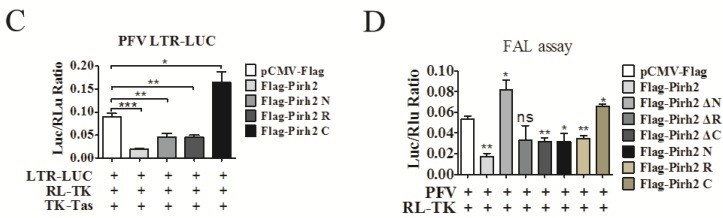
Identification of the key domain of Pirh2 that is responsible for the suppression of PFV. (**A**) Different domains of Pirh2 were cloned into the eukaryotic expression vector pCMV-3Flag; (**B**,**C**) The influences of Pirh2 ΔN, Pirh2 ΔR, Pirh2 ΔC (**B**) and Pirh2 N, Pirh2 R, Pirh2 C (**C**) on LTR transcription were determined by dual luciferase assay. 293T cells were transfected with LTR-Luc in the presence TK-Tas combined with different truncated Pirh2 plasmids. RL-TK was used to normalize transfection efficiency. At 48 h post transfection, luciferase activities were measured; (**D**) The relative viral load in the presence of different Pirh2 truncations was analyzed using the FAL assay with a PFV indicator cell line. All the data are representative of three independent experiments with triplicate samples. Asterisks denote significant differences of samples compared with the pCMV-Flag transfected group. (Student’s *t*-test, * *p* < 0.05; ** *p* < 0.01; *** *p* < 0.001, ns = not significant).

Pirh2 contains three domains, an N-terminal domain (NTD), a RING domain (RING), and a C-terminal domain (CTD), which fold independently of each other [20]. To identify functional domains of Pirh2 that are essential for its inhibitory effect on PFV replication and transcription, we generated a series of Pirh2 expression constructs with various coding region truncations, including deletions of the NTD, RING, and CTD (Figure 4A). The influences of each mutant Pirh2 on LTR transcriptional activation and PFV viral load were tested in a luciferase assay. We found that deletion of the NTD (Pirh2 ΔN) and RING (Pirh2 ΔR) showed no inhibitory effect on LTR transactivity (Figure 4B), suggesting that the NTD plays an important role in the inhibitory effect of Pirh2. Interestingly, Pirh2 ΔN was able to enhance the LTR transcriptional activation by 1.5-fold (Figure 4B). In addition, deletion of the CTD (Pirh2 ΔC) reduced LTR transcriptional activation by approximately 70%, which is similar to the complete Pirh2 (Figure 4B), suggesting that the CTD may not be required for Pirh2 to suppress the LTR. Taken together, the NTD and/or RING of Pirh2 may be important for reducing LTR transcription and viral replication inhibition.

Subsequently, to confirm whether a single domain of Pirh2 was sufficient to repress PFV LTR transcription, Pirh2 N, Pirh2 R, and Pirh2 C were transfected into 293T cells with PFV LTR-Luc. These results indicated that both the NTD and the RING of Pirh2 are capable of decreasing LTR transcription by over 50% compared to the control (Figure 4C). Because the RING domain of Pirh2 is associated with its E3 ubiquitin ligase activity, we speculated that ubiquitination may be involved in Pirh2-mediated inhibition. Interestingly, the Pirh2 CTD modestly promoted LTR transcription by 1.5-fold (Figure 4C). Based on the previous results and the Pirh2 ΔN results (Figure 4B), we hypothesized that the CTD may function as a transactivation domain.

Furthermore, the FAL assay showed similar effects of different Pirh2 mutants on PFV replication (Figure 4D). Deletion of the NTD or RING of Pirh2 did not inhibit PFV replication, and separately, the NTD and RING were capable of impairing PFV replication. In contrast, Pirh2ΔN and Pirh2C, which both contain the CTD, modestly facilitated PFV replication.

### 3.5. The E3 Ubiquitin Ligase Activity of Pirh2 Contributes to Its Inhibitory Effect on PFV

**Figure 5:**
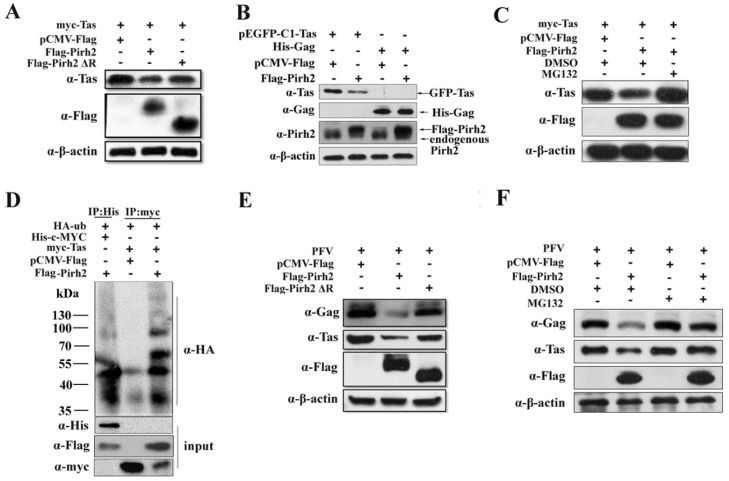
Pirh2 decreases the expression of Tas protein and inhibits PFV replication through ubiquitin-proteasome pathway. (**A**,**E**) Tas expression or PFV replication are down regulated by overexpressed Pirh2 and deletion of RING domain abrogates its inhibitory effect; (**B**) Pirh2 down regulates Tas specifically. 293T cells were transfected Flag-Pirh2 with pEGFP-C1-Tas or His-Gag, and Western blot was used to detect the expression of Tas or Gag; (**C**) Pirh2 down regulates the expression of Tas protein and the inhibition was reversed by MG132. myc-Tas combined with Flag-Pirh2 or pCMV-Flag (as control) were transfected into 293T cells; 8 h before harvest, the cells were treated with DMSO or MG132 (5 μM). Western blot was performed to assess Tas protein levels; (**D**) Pirh2 promotes Tas polyubiquitination. HA-tagged ubiquitin (HA-Ub), myc-Tas or c-MYC combined with Pirh2 were transfected into 293T cells, pCMV-Flag used as negative control and c-MYC used as positive control; (**F**) MG132 rescues the suppression of PFV produced by Pirh2. 293T cells were transfected with Flag-Pirh2 or pCMV-Flag (as control) for 24 h and challenged with PFV for another 48 h; 8 h before harvest, the cells transfected with Flag-Pirh2 or not were treated with DMSO or MG132 (5 μM). Then, Western blot was performed to assess the viral protein Gag and Tas.

Because the RING domain of Pirh2 is related to the ubiquitin-proteasome, we next investigated whether Pirh2 could influence the stability of the Tas protein. myc-Tas was co-transfected with Flag-Pirh2 or Flag-Pirh2ΔR, and pCMV-Flag was used as control. As shown in Figure 5A, overexpression of Pirh2 led to a decline in the level of Tas protein compared to the control. To demonstrate that Pirh2 overexpression are specific to down regulated Tas. 293T cells were transfected Flag-Pirh2 with pEGFP-C1-Tas or His-Gag, and Western blot was used to detect the expression of Tas or Gag. The result showed that overexpressed Pirh2 could down regulate pEGFP-C1-Tas but not the His-Gag (Figure 5B). And Pirh2 lacking the RING domain had little effect on Tas expression, suggesting that the inhibitory effect of Pirh2 on PFV is partially attributable to its E3 ubiquitin ligase activity. To further confirm whether the molecular mechanism is related to the proteasome degradation pathway, Pirh2-overexpressing cells were treated with the proteasome inhibitor MG132 at 5 μM for 8 h before lysis, and we found that Tas expression was notably rescued by MG132 (Figure 5C), indicating that Pirh2 may decrease Tas through the ubiquitin-proteasome pathway. Then, we performed an *in vivo* ubiquitination assay. The 293T cells were transfected with HA-tagged ubiquitin (HA-Ub), myc-Tas or c-MYC combined with Pirh2, pCMV-Flag used as a negative control, or c-MYC used as a positive control [28].

Immunoprecipitation was used with anti-myc or anti-His antibodies, and the precipitates were analyzed with anti-HA antibody. Overexpression of Pirh2 was found to significantly increased myc-Tas polyubiquitination, which was similar to c-MYC, indicating that Pirh2 polyubiquitinated Tas and mediated its proteasomal degradation (Figure 5D). We further tested by Western blot whether deletion of the RING domain or treatment with MG132 would reverse the inhibitory effect of Pirh2 on PFV replication. As expected, the PFV structural protein Gag and the regulatory protein Tas were slightly decreased by Pirh2ΔR compared to Pirh2 (Figure 5E), and MG132 was able to counteract the inhibitory effect of overexpressed Pirh2 (Figure 5F), but could not increase the Tas and Gag levels by itself. All of these results indicate that Pirh2 negatively influences viral replication by promoting the instability of Tas through the ubiquitin pathway.

## 4. Discussion

In this paper, we identified the human endogenous protein Pirh2 as a novel inhibitor of PFV replication. We found that PFV transactivator Tas is targeted by Pirh2 for the proteasome-dependent degradation, which results in the downregulations of the Tas-dependent transcriptional activation of the viral LTR and IP promoters. In addition, our results showed that RING is important for its anti-viral function as expect, and the NTD is also involved in.

The NTD and RING domain of Pirh2 were separately able to inhibit PFV LTR transcription and viral replication (Figure 4C,D). Because the RING is essential for the ubiquitin ligase activity of Pirh2, however, the interesting question was how does the NTD alone inhibit PFV replication? Although it has been reported that Pirh2 NTD is a cysteine-rich zinc finger domain, which is the molecular basis for direct binding to DNA, Pirh2 has never been shown to be a DNA binding protein [24]. Although NTD has been reported to interact with the p53 DNA binding domain [24], we speculated that NTD may interact with other proteins and further hinder Tas binding to the LTR and IP promoters to finally interfere with viral transcription and replication. The CTD was nonessential for the Pirh2 inhibitory effect on PFV LTR transcription and viral replication in our experiments (Figure 4C,D). Conversely, the CTD alone modestly promoted LTR transcription and viral replication (Figure 4C,D). Why does the CTD have the opposite effect? Residues 240–250 in the CTD of Pirh2 are required for Pirh2 self-ubiquitination and impact the stability of Pirh2 [31], which may indirectly facilitate the stability of Tas or increase the affinity of Tas for the LTR. 

As an E3 ubiquitin ligase, the most recognized function of Pirh2 is the participation in tumor genesis via regulating the stability of both oncogenes and tumor suppressor genes through the ubiquitin-proteasome pathway [20]. Interestingly, Pirh2 has also been reported to interact with ORF3 of porcine circovirus 2 (PCV2), resulting in the facilitation of p53 expression and the apoptosis of virally infected cells, which further impacted the viral pathogenicity of PCV2 indirectly [32,33]. In addition, Pirh2 was demonstrated to interact with CDK9, which also led to the facilitation of p53 expression, the stalling of transcriptional elongation of the HIV-1 LTR and a significant reduction in HIV-1 replication [34]. Recently, Pirh2 was also demonstrated to interact with measles virus phosphor protein (MV P) with no effect on MV replication. In our study, we found that Pirh2 could directly inhibit viral replication, possibly by interacting with Tas and ubiquitinating the viral protein Tas. Therefore, this was the first time that Pirh2 was shown to be an inhibitor of viral replication by directly interacting with a viral protein.

Because Tas is essential for viral replication, several host factors have been reported to interact with Tas and then impact viral transcription through different mechanisms [17,18,19]. For instance, the interaction between PML and Tas prevented Tas binding to the LTR and IP [17], and the interaction between N-Myc interactor (Nmi) and Tas facilitated the relocation of Tas from the nucleus to the cytoplasm [19]. Both of these interactions led to an inhibition of viral transcription, but the association of PCAF and Tas resulted in acetylation of Tas and enhanced its promoter-binding affinity and viral transcription [18,35]. In our study, the novel PFV inhibitor Pirh2 functioned by ubiquitin-degrading the transactivator Tas and was negatively involved in viral transcription. The ubiquitin-proteasome pathway is an important cellular process that is also involved in the life cycle of many viruses. Evidence has shown that the ubiquitin-proteasome pathway regulates the replication of HIV [36], PVC2 [37] and Coxsackievirus B3 [38]. This is the first report that the ubiquitin-proteasome pathway is involved in the regulation of PFV replication.

## 5. Conclusions

In conclusion, we identified the cellular factor Pirh2 as a novel inhibitor of PFV replication. Pirh2 was demonstrated to inhibit PFV replication and downregulate the Tas-dependent transcriptional activation of the viral LTR and IP promoters. Furthermore, it acts as an inhibitor by interacting with Tas and reducing the Tas protein level through ubiquitin-proteasome pathway. In addition, the viral inhibitory function of Pirh2 is N-terminal and RING domain dependent.

## Supplementary Files

Supplementary File 1
