# Supplementary material for: Human Pirh2 is A Novel Inhibitor of Prototype Foamy Virus Replication
Source: Viruses. 2015 Apr 2;7(4):1668–84. doi: 10.3390/v7041668 (PMC4411673; doi:10.3390/v7041668)
Supplement: Supplementary File 1 [file viruses-07-01668-s001.pdf]

Supplementary Information

Table S1. All the primers used for plasmid construction in this paper.

	Primers	Application
U-KpnI	5'-CTCGGTACCATCAGAACATTGA-3'	for LTR-Luc
D-XhoI	5'-TCACTCGAGAGCGAGTAGTGAAG-3'	
U-SmaI	5'-GATCCCGGGATATGTTCCTAGCATCGTGAC-3'	for IP-Luc
D- NcoI	5'AATCCATGGTACAATCTTAAATATAAGAATAACC-3'	
U-Bgl II	5'-GCCAGATCTATGGATTCCTACGAAAAAG-3'	for pEGFP-C1-Tas
D-KpnI	5'-CGGGGTACCTTATAAAACTGAATGTTCAC-3'	
U-SalI	5'-GGTCGACGATGGATTCCTACGAAAAAG-3'	for myc-Tas
D-KpnI	5'-GCGGTACCTTATAAAACTGAATGTTCACC-3'	
U-EcoRI	5'-AAGAATTCATGGCGGCGACGGCCCGGG-3'	for pDsRd-n1-Pirh2
D-kpnI	5'-CCGGTACCACTTGCTGATCCAGTGAAAT-3'	
U-EcoRI	5'-AAGAATTCATATGGCGGCGACGGCCCGG-3'	for Flag-Pirh2
D-kpnI	5'-CCGGTACCTCATTGCTGATCCAGTGAA-3'	
U-EcoRI	5'-ATGAATTCTAGCCACCATGGAAAATGTGTCCCGAC-3'	for Flag-Pirh2 ΔN
D-XhoI	5'-GCGCTCGAGTCATTGCTGATCCAGTGAA-3'	
P1-EcoRI	5'-ATGAATTCTAGCCACCATGGAAAATGTGTCCCGAC-3'	for Flag-Pirh2 ΔR
P2	5'-GGTCATATCTAAAGCAATACACTTGTGTCTTC-3'	
P3	5'-AGACACAAGTGTATTGCTTTAGATATGACCAC-3'	
P4--XhoI	5'-GCGCTCGAGTCATTGCTGATCCAGTGAA-3'	
U-EcoRI	5'-ATGAATTCTAGCCACCATGGCGGCGACGGCCCG-3'	for Flag-Pirh2 ΔC
D-XhoI	5'-GCCCTCGAGTTAAGAGTGCATACATAATG-3'	
U-EcoRI	5'-ATGAATTCTAGCCACCATGGCGGCGACGGCCCG-3'	for Flag-Pirh2 N
D-XhoI	5'-CATCTCGAGTCAAATACACTTGTGTCTTC-3'	
U-EcoRI	5'-ATGAATTCTAGCCACCATGGAAAATGTGTCCCGAC-3'	for Flag-Pirh2 R
D-XhoI	5'-CCGCTCGAGTCAAGAGTGCATACATAATGG-3'	
U-EcoRI	5'-ATGAATTCTAGCCACCATGGCTTTAGATATGACC-3'	for Flag-Pirh2 C
D-XhoI	5'-GCGCTCGAGTCATTGCTGATCCAGTGAA-3'	

© 2015 by the authors; licensee MDPI, Basel, Switzerland. This article is an open access article distributed under the terms and conditions of the Creative Commons Attribution license (http://creativecommons.org/licenses/by/4.0/).
